# Association of serum vitamin D level and live birth rate in women undergoing frozen embryo transfer—a retrospective cohort study

**DOI:** 10.1007/s10815-024-03326-z

**Published:** 2025-01-09

**Authors:** Jennifer K.Y. Ko, Mei Ting Lam, Kevin K.W. Lam, Tat On Chan, Raymond H.W. Li, Ernest H.Y. Ng

**Affiliations:** 1 Department of Obstetrics and Gynaecology, Li Ka Shing Faculty of Medicine, School of Clinical Medicine, The University of Hong Kong, Hong Kong, China; 2 Department of Obstetrics and Gynaecology, Kwong Wah Hospital, Hong Kong, China

**Keywords:** Vitamin D, Live birth rate, Pregnancy rate, Frozen embryo transfer, Retrospective cohort

## Abstract

**Purpose:**

To assess the association of serum vitamin D level and the live birth rate in women undergoing frozen embryo transfer (FET).

**Methods:**

This is a retrospective cohort study involving 1489 infertile women who had frozen embryo transfer at two tertiary reproductive medicine centres from 2019 to 2021. Only the first frozen embryo transfer was included for women who had repeated transfers during the period. Archived serum samples taken at LH surge or before the start of progestogens for FET were analysed for 25(OH)D levels using mass spectrometry. The main outcome measure was the live birth rate. Vitamin D deficiency was defined as serum 25(OH)D < 50 nmol/l (< 20 ng/ml) based on the Endocrine Society Clinical Practice guidelines.

**Results:**

The median age was 36 (25th–75th percentile 34–38) years. 37.7% (561/1489) women had cleavage stage embryo transfer and 62.3% (928/1489) women had blastocyst transfer. When analysing the results based on the threshold in the Endocrine Society guideline of 50 nmol/l (20 ng/ml) for vitamin D deficiency, there were no statistically significant differences in the live birth rate in the vitamin D deficient and non-deficient groups [151/489 (30.9%) vs 341/998 (34.2%), OR 0.861, 95% CI 0.683–1.086 *P* = 0.205]. There were no statistically significant differences in the pregnancy rates, ongoing pregnancy rates, and miscarriage rates between the two groups.

**Conclusion:**

Serum vitamin D is not associated with birth rate in women undergoing FET.

## Introduction

Vitamin D deficiency is a ‘global pandemic’ that affects all populations including reproductive-age women [1, 2]. While vitamin D is well known to be important for bone health, vitamin D receptors and vitamin D metabolizing enzymes have also been found in various reproductive organs including the ovaries, uterus, endometrium, and placenta and thus postulated to play a role in normal reproductive function [3].

Basic science studies have shown that vitamin D can affect ovarian follicular development and luteinization by altering AMH signalling, FSH sensitivity, and progesterone production and release in the human granulosa cells [4]. Other studies have suggested a possible effect of vitamin D on endometrial receptivity via regulating myometrial contraction and the expression of the homeobox gene HOXA10, which is a well-known molecule involved in the mechanism of implantation [5, 6]. In addition, vitamin D receptors have been found in maternal decidua and foetal trophoblast in early pregnancy, suggesting a role in trophoblast invasion, placental spiral artery remodelling, and regulating immune cell function [7, 8]. There is still controversy on how these mechanisms translate exactly to affect clinical reproductive outcome.

In vitro fertilization (IVF) provides a good model to study the individual steps in human reproduction. In recent years, meta-analyses have generally shown that vitamin D deficiency in women undergoing IVF was associated with a lower live birth rate compared to women who were vitamin D replete, although data is still not consistent [9–12]. Many of the studies assessed the vitamin D levels and pregnancy outcome at the fresh IVF cycle. High oestradiol levels during ovarian stimulation for IVF can stimulate increase in vitamin D-binding protein and affect the total vitamin D serum concentrations [13]. Moreover, embryo cryopreservation has become an integral part of modern-day IVF and many women may have all embryos frozen without fresh embryo transfer due to different clinical indications [14]. A retrospective study performed at our unit showed that the cumulative live birth rate of the first IVF cycle in the vitamin D deficient group was significantly lower compared to the non-deficient group [15]. However, serum vitamin D levels were assessed at the start of ovarian stimulation for the IVF cycle in our study and frozen embryo transfer could have occurred months later in some individuals.

Studies of IVF using donor oocytes enable factors that impact implantation (recipient factors) to be analysed separately from factors that influence ovarian stimulation and embryo quality (donor factors). Women who donate oocytes are presumably younger and have good oocyte quality, therefore the studied effect on recipients may be more related to endometrial factors. One retrospective cohort study involving 99 recipients of fresh embryo transfer from donor oocytes showed improvement in pregnancy rate in recipient women who were vitamin D replete compared to those who were deficient (live birth rates 59% vs 31%) and supported that the effect of vitamin D may be mediated via enhancing endometrial receptivity [16]. However, three other studies did not show any differences between pregnancy outcome in vitamin D deficient and replete recipients of embryos from donor oocytes [17–19] so the effect of vitamin D on endometrial receptivity is still controversial.

Only a few reports have studied the association of vitamin D and pregnancy outcomes in frozen embryo transfer and the results are again conflicting. A retrospective study of 368 infertile women undergoing single frozen blastocyst transfer showed that vitamin D deficiency compromised pregnancy rates by 40% [20]. Franasiak et al. further attempted to control for embryonic factors in another retrospective cohort study by including only euploid blastocyst transfers and did not find the serum vitamin D status to be related to pregnancy outcome, although the population was a mix of 517 first fresh or cryopreserved embryo transfers [21]. A prospective observational cohort study involving 280 patients undergoing frozen embryo transfer also did not show that vitamin D deficiency affected pregnancy rates [22]. These studies involved mostly Caucasian population and serum vitamin D was assessed using immunoassay.

In this study, we assessed the association between serum vitamin D level and the live birth rate in women undergoing frozen embryo transfer.

## Materials and methods

This was a retrospective study carried out at 2 tertiary reproductive medicine centres in Hong Kong, namely the Centre of Assisted Reproduction and Embryology, The University of Hong Kong—Queen Mary Hospital and the Dr. Stephen Chow Chun—Kay Assisted Reproduction Centre, Kwong Wah Hospital. Clinical details and laboratory data were prospectively entered into a computerized database by designated embryologists and retrieved for analysis. To avoid transcription error, the data was routinely double-checked by a second designated embryologist. Subjects are identified by three identifiers (name, identification number, and date of birth), and checked against the treatment date. In case of missing data, the centralized Hospital Authority electronic patient record system that links up all public hospitals in Hong Kong and paper records were reviewed to obtain the missing data. The study was approved by the Institutional Review Board of the University of Hong Kong/Hospital Authority Hong Kong West Cluster and the Kowloon Central Cluster/Kowloon East Cluster Research Ethics Committee.

### Patients

Women included in this retrospective analysis were those who were undergoing frozen embryo transfer at the two IVF centres between 2019 and 2021. Those undergoing in vitro maturation, pre-implantation genetic testing, and whose archived serum sample could not be retrieved were excluded. Those who had repeated IVF or frozen embryo transfer cycles during the period were counted only for the earliest cycle and therefore the first frozen embryo transfer of the first IVF cycle in the study period was used for analysis where possible.

The details of the procedures for ovarian stimulation, oocyte retrieval, handling of the gametes, cryopreservation of the embryos, and frozen embryo transfer were previously described [15, 23].

### Ovarian stimulation and IVF

The gonadotrophin-releasing hormone (GnRH) antagonist, long GnRH agonist, or progestin-primed protocols were used for pituitary downregulation. Human menopausal gonadotrophin (HMG) or recombinant FSH was used for ovarian stimulation. In the GnRH antagonist protocol, women received ganirelix (Orgalutran®, NV Organon, The Netherlands) or cetrorelix (Cetrotide®, Merck, Germany) 250 µm daily starting from the sixth day of stimulation. In the long GnRH agonist protocol, the women received buserelin (Suprecur®, Hoechst, Frankfurt, Germany) nasal spray 150 µm four times a day starting from the mid-luteal phase of the cycle preceding the treatment cycle. In the progestin-primed ovarian stimulation protocol, 10 mg daily medroxyprogesterone acetate was given simultaneously with gonadotrophins at the start of stimulation [24]. The initial dose of stimulation was determined according to the baseline antral follicle count (AFC). Human chorionic gonadotrophin (hCG) (Ovidrel® 250 µm) was given when the mean diameter of the leading follicle reached 18 mm and more than 3 follicles reaching a mean diameter of 16 mm or above, followed by transvaginal ultrasound-guided oocyte retrieval 36 h later. For women with excessive ovarian response using the GnRH antagonist or progestin-primed ovarian stimulation protocols as shown by serum oestradiol level of > 25,000 pmol/L, more than 15 follicles larger than 14 mm in diameter or evidence of ovarian hyperstimulation syndrome, GnRH agonist (Decapeptyl®, Ferring, Kiel, Germany) 0.3 mg can be given subcutaneously to replace hCG to trigger final oocyte maturation. Fertilization was carried out either by conventional insemination or intracytoplasmic sperm injection (ICSI) depending on semen parameters.

### Cryopreservation of embryos

Prior to June 2020, cryopreservation of day 2 cleavage stage embryos was performed by a slow freezing protocol using a programmable freezer (Planer Products Ltd.; Sunbury-On-Thames, UK) in Queen Mary Hospital. Vitrification was performed for cleavage-stage embryos in Kwong Wah Hospital and starting from June 2020 in Queen Mary Hospital as well. Vitrification was performed for blastocysts in both hospitals. Extended culture to blastocyst was advised if there were more than 3 good quality cleavage stage embryos 2 days after retrieval.

### Endometrial preparation in frozen embryo cycles

Frozen embryo transfer was performed in natural, letrozole-induced or hormone replacement cycles. Frozen embryos or blastocysts were transferred in natural cycles in ovulatory women and in letrozole-induced or hormone-replacement cycles for anovulatory women. Hormone replacement cycles can also be used in ovulatory women who had difficulty in identifying the day of LH surge in previous cycles or for scheduling issues.

In natural cycles, women had a daily blood test to identify the day of LH surge starting from 18 days before the next expected period (17). The LH surge was defined by the LH level being above 20 IU/L and more than double the average over the past 3 days. Luteal phase support was not routinely given.

In letrozole-induced cycles, women received letrozole 2.5 mg daily for 5 days, followed by daily monitoring of serum LH, similar to that in natural cycles. Letrozole was stepped up to 5–7.5 mg if needed. Luteal phase support was not routinely given.

In hormone replacement cycles, women received oral oestradiol 6 mg daily for 14 days for endometrial priming, followed by transvaginal ultrasonography for endometrial thickness and addition of vaginal micronized progesterone 100 mg and oral dydrogesterone 10 mg three times daily if endometrial thickness reached 7 mm or above. If they were pregnant, these women continued oral oestradiol and vaginal/oral progestogen after frozen embryo transfer until 12 weeks of gestation.

For natural cycles and letrozole-induced cycles, frozen day 2 or day 3 embryos were transferred on the third or fourth day respectively after the LH surge. For hormone replacement cycles, frozen day 2 or day 3 embryos were transferred on the fourth or fifth day after starting progestogen. Frozen blastocysts were transferred on the sixth day after the LH surge or on the seventh day after starting progestogen.

Women were allowed to have a replacement of at most two cleavage stage embryos if she was > 38 years old or had failed 2 previous IVF cycles, and had no live birth, but single embryo transfer was strongly encouraged otherwise. Only single blastocyst transfer was allowed.

### Embryo transfer procedure

The embryo transfer was performed under transabdominal ultrasound guidance with a soft catheter (Sydney IVF Embryo Transfer Catheter®, Cook, Indiana, USA).

### Follow up

Urine pregnancy test was performed 18 days after the LH surge in FET cycles. If the urine pregnancy test was positive, transvaginal scanning was performed at 6 and 8 weeks of gestation to confirm foetal viability and ongoing pregnancy. Pregnancy outcomes were tracked from the Hospital Authority electronic patient record system or self-returned reply slips from the women or their obstetricians. If the woman did not deliver within the public hospital system and no reply letter was received 2–3 months after the expected date of delivery, they were contacted by our nurses to update the database.

### Serum 25-OH vitamin D measurement

Archived serum samples used for analysis in this study were taken at luteinizing hormone (LH) surge in natural or letrozole-induced cycles or 14 days after the start of oestradiol and at the commencement of progestogens in hormone replacement cycles. Residual serum after the routine clinical tests was archived at − 20 °C with written informed consent from the patients for research purposes anonymously. The average time was around 4 h between drawing the sample and archiving. The archived serum samples were retrieved and assayed for serum 25(OH)D concentration using liquid chromatography-mass spectrometry (LC–MS) at Adicon Clinical Laboratories Limited, which is accredited by the Chinese National Center for Clinical Laboratories (NCCL), External Quality Assessment (EQA), and Trueness Verification of 25(OH)D Assays scheme. The limit of quantification is 2.5 nmol/l (1 ng/ml). The within-run and between-run trueness for low, medium, and high levels of vitamin D2 are 92.0–113.2%, 94.3–106.7%, and 100.4–106.8%, respectively, whereas that for vitamin D3 are 104.7–118.5%, 97.8–109.6%, and 103.7–110.3% respectively. The co-efficient of variation within and between-runs for low, medium, and high levels of vitamin D2 are 4.38–6.05%, 2.24–4.17%, and 1.63–2.03%, respectively, and for vitamin D3 are 0.86–6.33%, 1.12–3.55%, and 0.72–2.85%.

Vitamin D deficiency was defined as serum 25(OH)D levels < 50 nmol/L (< 20 ng/ml) and vitamin D insufficiency as serum 25(OH)D ≥ 50 and < 75 nmol/L (≥ 20 and < 30 ng/ml) in accordance with the Endocrine Society criteria [25]. Serum 25(OH)D levels of ≥ 75 nmol/L (30 ng/ml) were considered replete. Women who were vitamin D insufficient and replete were grouped together as the non-deficient group and compared with those who were vitamin D deficient in the primary analysis. The three groups (vitamin D deficient, insufficient, and replete) were separately compared in further analysis.

### Main outcomes

The primary outcome was the live birth rate. Live birth was defined as the delivery of an infant born alive after 24 weeks of gestation (the definition of foetal viability adopted in this locality). Other outcomes included the pregnancy rate, ongoing pregnancy rate, multiple pregnancy rate, miscarriage rate, and ectopic pregnancy rate. Ongoing pregnancy was defined as a viable pregnancy beyond 8 weeks of gestation. Miscarriage was defined as the spontaneous loss of clinical pregnancy before 20 completed weeks of gestational age. Ectopic pregnancy was defined as a pregnancy outside the uterine cavity, diagnosed by ultrasound, surgical visualization, or histopathology.

### Statistical tests

Data were entered and analysed using IBM SPSS 26.0 (IBM Corporation, NY, USA). Continuous and categorical variables were compared between groups using the Mann–Whitney *U* test, Kruskal–Wallis test, and Chi-square test respectively. Odds ratio was calculated with the vitamin D non-deficient group as the reference group. Logistic regression analysis was performed to assess the effect of vitamin D on pregnancy outcomes controlling for confounders including women’s age at oocyte retrieval, body mass index, antral follicle count, stage of embryos in frozen transfer, type, and duration of infertility. The two-tailed *P* value of < 0.05 was considered statistically significant.

## Results

**Fig. 1:**
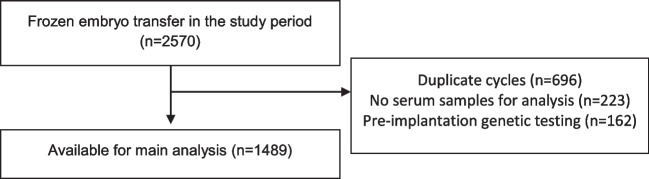
Flowchart of participants

One thousand four hundred eighty-nine women who had FET during the study period were available for analysis. The flow of participants is shown in Fig. 1. For women who had repeat frozen embryo transfer cycles in the study period, only the first frozen embryo transfer was counted and the other duplicate cycles were excluded.

The median age of the women at oocyte retrieval was 36 (25th–75th percentile 34–38) years. A total of 561/1489 (37.7%) women had cleavage stage embryo transfer and 928/1489 (62.3%) women had blastocyst transfer. A total of 1482/1489 (99.5%) women had single cleavage stage or blastocyst transfer and 7 (0.5%) women had 2 cleavage-stage embryos transferred. A total of 896/1489 (60.2%) had natural cycles, 408/1489 (27.4%) had hormone replacement cycle, 184/1489 (12.4%) had letrozole-induced cycle and 1/1489 (0.1%) had stimulated cycle frozen embryo transfer.

The median serum 25(OH)D was 58.7 (25th–75th percentile 45.7–75.0] nmol/l (23.5 ng/ml), where 489/1489 (32.8%) and 1000/1489 (67.2%) women were vitamin D deficient (< 50 nmol/l or < 20 ng/ml) and non-deficient (≥ 50 nmol/l or ≥ 20 ng/ml), respectively. Using the Endocrine Society Guidelines criteria, 489/1489 (32.8%), 627/1489 (42.1%), and 373/1489 (25.1%) women were vitamin D deficient (< 50 nmol/l or < 20 ng/ml), insufficient (≥ 50 nmol/l and < 75 nmol/l or ≥ 20 ng/ml and < 30 ng/ml) and sufficient (≥ 75 nmol/l or ≥ 30 ng/ml), respectively.

The patient characteristics in the vitamin D deficient and non-deficient groups are shown in Table 1. There were no significant differences in the women’s age, duration, type, and cause of infertility and antral follicle count between the two groups. Women who were vitamin D deficient had a significantly higher body mass index compared to the non-deficient group although the difference was small [22.7 (20.6–25.4) vs. 21.9 (20.2–24.0), *P* = < 0.001]. A higher proportion of women in the vitamin D non-deficient group had blastocyst transfer compared to the deficient group. The median (25th–75th percentile) vitamin D level in women with cleavage-stage embryos and blastocysts were 57.5 (42.4–75.7) nmol/l and 59.4 (47.1–74.5) nmol/l, respectively, which was not significantly different (*P* = 0.133).

**Table 1:** Patient characteristics

Parameters	Vitamin D deficient group < 50 nmol/l (< 20 ng/ml) (*n* = 489)	Vitamin D non-deficient group ≥ 50 nmol/l (≥ 20 ng/ml) (*n* = 1000)	*P* values^#^
Index stimulated IVF cycle			
Age of women at oocyte retrieval (year)	36 (34–38)	36 (34–38)	0.676
Body mass index (kg/m2)	22.7 (20.6–25.4)	21.9 (20.2–24.0)	< 0.001*
Non-smoker	440/487 (90.3)	905/984 (92.0)	0.515
Ethnicity			< 0.001*
Chinese	446 (91.2)	969 (96.9)	
Non-Chinese	43 (8.8)	31 (3.1)	
Duration of infertility (years)	4.0 (3.0–6.0)	4.0 (3.0–6.0)	0.426
Cause of infertility n (%)			0.746
Endometriosis	33 (6.7)	50 (5.0)	
Tuboperitoneal factor	58 (11.9)	121 (12.1)	
Male factor	171 (35.0)	356 (35.6)	
Unexplained	117 (23.9)	240 (24.0)	
Mixed factors	110 (22.5)	233 (23.3)	
Type of infertility			0.201
Primary	357 (73.0)	698 (69.8)	
Secondary	132 (27.0)	302 (30.2)	
Antral follicle count	11 (7–16)	11 (7–16)	0.923
Stimulation regimen			0.370
Long GnRH agonist	5 (1.0)	7 (0.7)	
GnRH antagonist	459 (93.9)	925 (92.5)	
Progestin primed	25 (5.1)	68 (6.8)	
Total dose of gonadotrophins (IU)	2700 (2025–3300)	2475 (2100–3150)	0.386
Duration of stimulation (days)	11 (10–12)	11 (10–12)	0.044*
Peak estradiol level (pmol/l)	12,750 (8026–21,333)	12,840 (8015–20,357)	0.921
Number of oocytes retrieved	12 (7–17)	11 (7–16)	0.258
Number of oocytes normally fertilized	7 (4–10)	6 (4–10)	0.767
Number of cleavage stage embryos in IVF cycle (*n* = 561)	3 (2–3)	3 (2–3)	0.533
Number of blastocysts in IVF cycle (*n* = 928)	4 (3–6)	4 (3–6)	0.248
Endometrial thickness on trigger day (mm)	11.2 (9.7–12.7)	11.1 (9.5–13.0)	0.877
Stage of embryos in frozen transfer			0.018*
Cleavage stage embryos Blastocysts	205 (41.9) 284 (58.1)	356 (35.6) 644 (64.4)	
Blastulation rate (only for women undergoing extended culture)	50% (33.3%–66.7%) (*n* = 284)	50% (33.3%–66.7%) (*n* = 644)	0.764
Number of embryos transferred			1.000 (Fisher’s exact test)
1	203 (99.0)	351 (98.6)	
2	2 (1.0)	5 (1.4)	
Number of blastocysts transferred			
1	284 (100.0)	644 (100.0)	–-
Endometrial preparation			0.138
Natural cycle	310 (63.4)	586 (58.6)	
Hormonal replacement cycle	116 (23.7)	292 (29.2)	
Letrozole	63 (12.9)	121 (12.1)	
Gonadotrophin stimulation	0 (0.0)	1 (0.1)	

Data shown represent the median (25th–75th percentile) for continuous variables and number (%) for categorical variables

^*^Statistically significant

### Main outcomes

The main outcomes are shown in Table 2. When analysing the results based on the threshold in the Endocrine Society guideline of 50 nmol/l (20 ng/ml) for vitamin D deficiency, there were no statistically significant differences in live birth rate, pregnancy rate, ongoing pregnancy rate, and miscarriage rate between the vitamin D deficient and non-deficient groups. The live birth rate, pregnancy rate, ongoing pregnancy rate, and miscarriage rate between the vitamin D deficient and non-deficient groups remained insignificant after adjusting for women’s age at oocyte retrieval, body mass index, antral follicle count, stage of embryos in frozen transfer, type, and duration of infertility.

**Table 2:** Pregnancy outcomes of frozen embryo transfer in the vitamin D deficient group and non-deficient group

	**Vitamin D deficient group** ** < 50 nmol/l (< 20 ng/ml)**	**Vitamin D non-deficient group** ** ≥ 50 nmol/l (≥ 20 ng/ml)**	**Odds ratio (95% CI)**	***P* values**	**Adjusted odds ratio (95% CI)#**	**Adjusted *P* values**
Pregnancy rate	208/489 (42.5%)	468/1000 (46.8%)	0.842 (0.677–1.046)	0.121	0.888 (0.705–1.118)	0.312
Ongoing pregnancy rate	153/489 (31.3%)	361/1000 (36.1%)	0.806 (0.640–1.015)	0.067	0.844 (0.662–1.076)	0.171
Live birth rate	151/489 (30.9%)	341/998 (34.2%)	0.861 (0.683–1.086)	0.205	0.905 (0.710–1.155)	0.423
Miscarriage rate	53/208 (25.5%)	120/468 (25.6%)	0.992 (0.682–1.443)	0.965	0.964(0.657–1.414)	0.852
Ectopic pregnancy	3/208 (1.4%)	2/468 (0.4%)	3.413 (0.566–20.408)	0.173^@^	3.025 (0.483–18.924)	0.237
Stillbirth	–	1/468 (0.2%)	–	1.000^@^	–	–
Multiple pregnancy	2/208 (1.0%)	6/468 (1.3%)	0.747 (0.150–3.731)	1.000^@^	0.677 (0.134–3.412)	0.636

Data shown represent the median (25th–75th percentile) or number (%)

^#^Adjusted for age of women at oocyte retrieval, body mass index, antral follicle count, stage of embryos in frozen transfer, type and duration of infertility

^@^Fisher’s exact test

1 woman in the vitamin D deficient group and 2 women in the vitamin D non-deficient group had termination of pregnancy after ongoing pregnancy. Another 2 women in the vitamin D non-deficient group were loss to follow-up after documented ongoing pregnancy, one of whom was last seen at the antenatal clinic at 28 weeks and subsequently emigrated abroad

When vitamin D insufficiency was analysed among deficient, insufficient, and sufficient groups, there were no statistically significant differences in the live birth rates [151/489 (30.9%), 218/627 (34.8%), 123/371 (33.2%), *P* = 0.391] among the vitamin D deficient, insufficient and sufficient groups, respectively. No statistically significant differences were found in the pregnancy rates, ongoing pregnancy rates, and miscarriage rates among the three groups (Table 3).

**Table 3:** Pregnancy outcomes of frozen embryo transfer using the Endocrine Society Guidelines cut-off

	***n* (%)**	***P* values**^**@**^	**Adjusted odds ratio (95% CI)**^**#**^	**Adjusted *P* values**
Pregnancy rate		0.348		
Vitamin D deficient group < 50 nmol/l (< 20 ng/ml)	208/489 (42.5%)		0.893 (0.670–1.190)	0.439
Vit D insufficient group (≥ 50 nmol/L and < 75 nmol/L) (≥ 20 ng/ml and < 30 ng/ml)	299/627 (47.7%)		1.009 (0.769–1.323)	0.948
Vit D sufficient group (≥ 75 nmol/L) (≥ 30 ng/ml)	169/373 (45.3%)		Ref	–
Ongoing pregnancy rate		0.130		
Vitamin D deficient group < 50 nmol/l (< 20 ng/ml)	153/489 (31.3%)		0.809 (0.599–1.091)	0.165
Vit D insufficient group (≥ 50 nmol/L and < 75 nmol/L) (≥ 20 ng/ml and < 30 ng/ml)	227/627 (36.2%)		0.934 (0.706–1.236)	0.633
Vit D sufficient group (≥ 75 nmol/L) (≥ 30 ng/ml)	134/373 (35.9%)		Ref	–
Live birth rate		0.421		
Vitamin D deficient group < 50 nmol/l (< 20 ng/ml)	151/489 (30.9%)		0.906 (0.670–1.227)	0.524
Vit D insufficient group (≥ 50 nmol/L and < 75 nmol/L) (≥ 20 ng/ml and < 30 ng/ml)	218/627 (34.8%)		1.002 (0.754–1.330)	0.991
Vit D sufficient group (≥ 75 nmol/L) (≥ 30 ng/ml)	123/371 (33.2%)		Ref	–
Miscarriage rate		0.811		
Vitamin D deficient group < 50 nmol/l (< 20 ng/ml)	53/208 (25.5%)		1.024 (0.635–1.652)	0.922
Vit D insufficient group (≥ 50 nmol/L and < 75 nmol/L) (≥ 20 ng/ml and < 30 ng/ml)	79/299 (26.4%)		1.098 (0.707–1.707)	0.676
Vit D sufficient group (≥ 75 nmol/L) (≥ 30 ng/ml)	41/169 (24.3%)		Ref	–
Ectopic pregnancy		0.303		
Vitamin D deficient group < 50 nmol/l (< 20 ng/ml)	3/208 (1.4%)		2.082 (0.206–21.018)	0.534
Vit D insufficient group (≥ 50 nmol/L and < 75 nmol/L) (≥ 20 ng/ml and < 30 ng/ml)	1/299 (0.3%)		0.526 (0.032–8.540)	0.651
Vit D sufficient group (≥ 75 nmol/L) (≥ 30 ng/ml)	1/169 (0.6%)		Ref	–
Stillbirth		0.938		
Vitamin D deficient group < 50 nmol/l	–		–	–
Vit D insufficient group (≥ 50 nmol/L and < 75 nmol/L) (≥ 20 ng/ml and < 30 ng/ml)	1/299 (0.3%)		–	–
Vit D sufficient group (≥ 75 nmol/L) (≥ 30 ng/ml)	–		–	–
Multiple pregnancy		0.797		
Vitamin D deficient group < 50 nmol/l (< 20 ng/ml)	2/208 (1.0%)		1.539 (0.138–17.222)	0.726
Vit D insufficient group (≥ 50 nmol/L and < 75 nmol/L) (≥ 20 ng/ml and < 30 ng/ml)	5/299 (1.7%)		3.072 (0.353–26.758)	0.309
Vit D sufficient group (≥ 75 nmol/L) (≥ 30 ng/ml)	1/169 (0.6%)		Ref	–

Data shown represent the number (%)

^@^Chi-square test for trend

^#^Adjusted for age of women at oocyte retrieval, body mass index, antral follicle count, stage of embryos in frozen transfer, type and duration of infertility

Subgroup analysis on pregnancy outcome was performed for women who had single blastocyst transfer only and cleavage-stage embryos only respectively and no statistically significant differences were found in the pregnancy rates, ongoing pregnancy rates, live birth rates, and miscarriage rates between the vitamin D deficient vs vitamin D non-deficient groups or among the vitamin D deficient, insufficient and sufficient groups (Tables 4, 5, 6, and 7).

**Table 4:** Pregnancy outcomes of frozen embryo transfer in the vitamin D deficient group and non-deficient group (for those with single blastocyst transfer only)

	**Vitamin D deficient group** ** < 50 nmol/l** (< 20 ng/ml)	**Vitamin D non-deficient group** ** ≥ 50 nmol/l** (≥ 20 ng/ml)	**Odds ratio (95% CI)**	***P* values**	**Adjusted odds ratio (95% CI)#**	**Adjusted *P* values**
Pregnancy rate	156/284 (54.9%)	369/644 (57.3%)	0.908 (0.686–1.203)	0.502	0.896 (0.675–1.190)	0.449
Ongoing pregnancy rate	117/284 (41.2%)	292/644 (45.3%)	0.845 (0.637–1.121)	0.241	0.835 (0.628–1.112)	0.218
Live birth rate	116/284 (40.8%)	273/642 (42.5%)	0.934 (0.703–1.239)	0.633	0.924 (0.694–1.232)	0.590
Miscarriage rate	36/156 (23.1%)	90/369 (24.4%)	0.930 (0.598–1.447)	0.747	0.947 (0.605–1.483)	0.812
Ectopic pregnancy	3/156 (1.9%)	1/369 (0.3%)	7.194 (0.745–71.429)	0.081^@^	6.707 (0.666–67.539)	0.106
Stillbirth	–	1/369 (0.3%)	–	1.000^@^	–	–
Multiple pregnancy	2/156 (1.3%)	4/369 (1.1%)	1.185 (0.215–6.536)	1.000^@^	0.961 (0.171–5.398)	0.964

Data shown represent the median (25th–75th percentile) or number (%)

^#^Adjusted for age of women at oocyte retrieval, body mass index, antral follicle count, type and duration of infertility

^@^Fisher’s exact test

**Table 5:** Pregnancy outcomes of frozen embryo transfer using the Endocrine Society Guidelines cut-off (for those with single blastocyst transfer only)

	***n* (%)**	***P* values**^**@**^	**Adjusted odds ratio (95% CI)**^**#**^	**Adjusted *P* values**
Pregnancy rate		0.430		
Vitamin D deficient group < 50 nmol/l (< 20 ng/ml)	156/284 (54.9%)		0.843 (0.590–1.204)	0.348
Vit D insufficient group (≥ 50 nmol/L and < 75 nmol/L) (≥ 20 ng/ml and < 30 ng/ml)	237/418 (56.7%)		0.910 (0.654–1.267)	0.577
Vit D sufficient group (≥ 75 nmol/L) (≥ 30 ng/ml)	132/226 (58.4%)		Ref	–
Ongoing pregnancy rate		0.097		
Vitamin D deficient group < 50 nmol/l (< 20 ng/ml)	117/284 (41.2%)		0.723 (0.506–1.032)	0.074
Vit D insufficient group (≥ 50 nmol/L and < 75 nmol/L) (≥ 20 ng/ml and < 30 ng/ml)	182/418 (43.5%)		0.800 (0.576–1.111)	0.183
Vit D sufficient group (≥ 75 nmol/L) (≥ 30 ng/ml)	110/226 (48.7%)		Ref	–
Live birth rate		0.406		
Vitamin D deficient group < 50 nmol/l (< 20 ng/ml)	116/284 (40.8%)		0.840 (0.586–1.202)	0.340
Vit D insufficient group (≥ 50 nmol/L and < 75 nmol/L) (≥ 20 ng/ml and < 30 ng/ml)	173/418 (41.4%)		0.863 (0.620–1.202)	0.383
Vit D sufficient group (≥ 75 nmol/L) (≥ 30 ng/ml)	100/224 (44.6%)		Ref	–
Miscarriage rate		0.655		
Vitamin D deficient group < 50 nmol/l (< 20 ng/ml)	36/156 (23.1%)		1.168 (0.661–2.066)	0.592
Vit D insufficient group (≥ 50 nmol/L and < 75 nmol/L) (≥ 20 ng/ml and < 30 ng/ml)	63/237 (26.6%)		1.372 (0.819–2.299)	0.229
Vit D sufficient group (≥ 75 nmol/L) (≥ 30 ng/ml)	27/132 (20.5%)		Ref	–
Ectopic pregnancy		0.218		
Vitamin D deficient group < 50 nmol/l (< 20 ng/ml)	3/156 (1.9%)		2.055 (0.199–21.174)	0.545
Vit D insufficient group (≥ 50 nmol/L and < 75 nmol/L) (≥ 20 ng/ml and < 30 ng/ml)	–		–	–
Vit D sufficient group (≥ 75 nmol/L) (≥ 30 ng/ml)	1/132 (0.8%)		Ref	–
Stillbirth		0.951		
Vitamin D deficient group < 50 nmol/l (< 20 ng/ml)	–		–	–
Vit D insufficient group (≥ 50 nmol/L and < 75 nmol/L) (≥ 20 ng/ml and < 30 ng/ml)	1/237 (0.4%)		–	–
Vit D sufficient group (≥ 75 nmol/L) (≥ 30 ng/ml)	–		–	–
Multiple pregnancy		0.338		
Vitamin D deficient group < 50 nmol/l (< 20 ng/ml)	2/156 (1.3%)		–	–
Vit D insufficient group (≥ 50 nmol/L and < 75 nmol/L) (≥ 20 ng/ml and < 30 ng/ml)	4/237 (1.7%)		–	–
Vit D sufficient group (≥ 75 nmol/L) (≥ 30 ng/ml)	–		–	–

Data shown represent the number (%)

^@^Chi-square test for trend

^#^Adjusted for age of women at oocyte retrieval, body mass index, antral follicle count, stage of embryos in frozen transfer, type and duration of infertility

**Table 6:** Pregnancy outcomes of frozen embryo transfer in the Vitamin D deficient group and non-deficient group (for those with cleavage-stage embryos only)

	**Vitamin D deficient group** ** < 50 nmol/l (< 20 ng/ml)**	**Vitamin D non-deficient group** ** ≥ 50 nmol/l (≥ 20 ng/ml)**	**Odds ratio (95% CI)**	***P* values**	**Adjusted odds ratio (95% CI)#**	**Adjusted *P* values**
Pregnancy rate	52/205 (25.4%)	99/356 (27.8%)	0.882 (0.597–1.304)	0.530	0.884 (0.592–1.321)	0.549
Ongoing pregnancy rate	36/205 (17.6%)	69/356 (19.4%)	0.886 (0.568–1.383)	0.594	0.883 (0.557–1.397)	0.594
Live birth rate	35/205 (17.1%)	68/356 (19.1%)	0.872 (0.556–1.366)	0.550	0.875 (0.551–1.390)	0.573
Miscarriage rate	17/52 (32.7%)	30/99 (30.3%)	1.117 (0.543–2.299)	0.763	0.997 (0.471–2.109)	0.994
Ectopic pregnancy	–	1/99 (1.0%)	–	1.000^@^	–	–
Stillbirth	–	–	–	–	–	–
Multiple pregnancy	–	2/99 (2.0%)	–	0.545^@^	–	–

Data shown represent the median (25th–75th percentile) or number (%)

^#^Adjusted for age of women at oocyte retrieval, body mass index, antral follicle count, type and duration of infertility

^@^Fisher’s exact test

**Table 7:** Pregnancy outcomes of frozen embryo transfer using the Endocrine Society Guidelines cut-off (for those with cleavage-stage embryos only)

	***n* (%)**	***P* values**^**@**^	**Adjusted odds ratio (95% CI)**^**#**^	**Adjusted *P* values**
Pregnancy rate		0.941		
Vitamin D deficient group < 50 nmol/l (< 20 ng/ml)	52/205 (25.4%)		1.020 (0.619–1.682)	0.937
Vit D insufficient group (≥ 50 nmol/L and < 75 nmol/L) (≥ 20 ng/ml and < 30 ng/ml)	62/209 (29.7%)		1.267 (0.781–2.057)	0.338
Vit D sufficient group (≥ 75 nmol/L) (≥ 30 ng/ml)	37/147 (25.2%)		Ref	–
Ongoing pregnancy rate		0.875		
Vitamin D deficient group < 50 nmol/l (< 20 ng/ml)	36/205 (17.6%)		1.123 (0.626–2.014)	0.698
Vit D insufficient group (≥ 50 nmol/L and < 75 nmol/L) (≥ 20 ng/ml and < 30 ng/ml)	45/209 (21.5%)		1.479 (0.843–2.596)	0.173
Vit D sufficient group (≥ 75 nmol/L) (≥ 30 ng/ml)	24/147 (16.3%)		Ref	–
Live birth rate		0.851		
Vitamin D deficient group < 50 nmol/l (< 20 ng/ml)	35/205 (17.1%)		1.152 (0.638–2.080)	0.639
Vit D insufficient group (≥ 50 nmol/L and < 75 nmol/L) (≥ 20 ng/ml and < 30 ng/ml)	45/209 (21.5%)		1.562 (0.885–2.754)	0.124
Vit D sufficient group (≥ 75 nmol/L) (≥ 30 ng/ml)	23/147 (15.6%)		Ref	–
Miscarriage rate		0.701		
Vitamin D deficient group < 50 nmol/l (< 20 ng/ml)	17/52 (32.7%)		0.675 (0.270–1.687)	0.401
Vit D insufficient group (≥ 50 nmol/L and < 75 nmol/L) (≥ 20 ng/ml and < 30 ng/ml)	16/62 (25.8%)		0.522 (0.211–1.292)	0.160
Vit D sufficient group (≥ 75 nmol/L) (≥ 30 ng/ml)	14/37 (37.8%)		Ref	–
Ectopic pregnancy		0.896		
Vitamin D deficient group < 50 nmol/l (< 20 ng/ml)	–		–	–
Vit D insufficient group (≥ 50 nmol/L and < 75 nmol/L) (≥ 20 ng/ml and < 30 ng/ml)	1/62 (1.6%)		–	–
Vit D sufficient group (≥ 75 nmol/L) (≥ 30 ng/ml)	–		–	–
Stillbirth		–		
Vitamin D deficient group < 50 nmol/l (< 20 ng/ml)	–		–	–
Vit D insufficient group (≥ 50 nmol/L and < 75 nmol/L) (≥ 20 ng/ml and < 30 ng/ml)	–		–	–
Vit D sufficient group (≥ 75 nmol/L) (≥ 30 ng/ml)	–		–	–
Multiple pregnancy		0.264		
Vitamin D deficient group < 50 nmol/l (< 20 ng/ml)	–		–	–
Vit D insufficient group (≥ 50 nmol/L and < 75 nmol/L) (≥ 20 ng/ml and < 30 ng/ml)	1/62 (1.6%)		0.967 (0.039–23.679)	0.983
Vit D sufficient group (≥ 75 nmol/L) (≥ 30 ng/ml)	1/37 (2.7%)		Ref	–

Data shown represent the number (%)

^@^Chi-square test for trend

^#^Adjusted for age of women at oocyte retrieval, body mass index, antral follicle count, stage of embryos in frozen transfer, type and duration of infertility

The patient characteristics in the vitamin D deficient, insufficient, and replete groups are shown in Table 8.

**Table 8:** Patient characteristics in 3 groups

Parameters	Vitamin D deficient group < 50 nmol/l (< 20 ng/ml) (*n* = 489)	Vitamin D insufficient group ≥ 50 nmol/l and < 75 nmol/l (≥ 20 ng/ml and < 30 ng/ml) (*n* = 627)	Vitamin D sufficient group ≥ 75 nmol/l (≥ 30 ng/ml) (*n* = 373)	*P* values^#^
Index stimulated IVF cycle				
Age of women at oocyte retrieval (year)	36 (34–38)	36 (34–37)	36 (34–38)	0.705
Body mass index (kg/m2)	22.7 (20.6–25.4)	22.0 (20.2–24.3)	21.9 (20.3–24.3)	< 0.001*
Non-smoker	440/487 (90.3)	569/618 (92.1)	336/366 (91.8)	0.820
Ethnicity				< 0.001*
Chinese	446 (91.2)	612 (97.6)	357 (95.7)	
Non-Chinese	43 (8.8)	15 (2.4)	16 (4.3)	
Duration of infertility (years)	4.0 (3.0–6.0)	4.0 (3.0–6.0)	4.0 (3.0–6.0)	0.175
Cause of infertility *n* (%)				0.801
Endometriosis	33 (6.7)	32 (5.1)	18 (4.8)	
Tuboperitoneal factor	58 (11.9)	77 (12.3)	44 (11.8)	
Male factor	171 (35.0)	217 (34.6)	139 (37.3)	
Unexplained	117 (23.9)	160 (25.5)	80 (21.4)	
Mixed factors	110 (22.5)	141 (22.5)	92 (24.7)	
Type of infertility				0.440
Primary	357 (73.0)	437 (69.7)	261 (70.0)	
Secondary	132 (27.0)	190 (30.3)	112 (30.0)	
Antral follicle count	11 (7–16)	11 (7–16)	10 (7–15)	0.355
Stimulation regimen				0.269
Long GnRH agonist	5 (1.0)	5 (0.8)	2 (0.5)	
GnRH antagonist	459 (93.9)	573 (91.4)	352 (94.4)	
Progestin primed	25 (5.1)	49 (7.8)	19 (5.1)	
Total dose of gonadotrophins (IU)	2700 (2025–3300)	2475 (2138–3150)	2475 (2100–3150)	0.663
Duration of stimulation (days)	11 (10–12)	11 (10–12)	11 (10–12)	0.041*
Peak estradiol level (pmol/l)	12,750 (8026–21,333)	13,255 (8064–21,028)	12,350 (7911–17,968)	0.511
Number of oocytes retrieved	12 (7–17)	12 (7–17)	10 (7–15)	0.019*
Number of oocytes normally fertilized	7 (4–10)	7 (4–10)	6 (4–9)	0.185
Number of cleavage stage embryos in IVF cycle (*n* = 561)	3 (2–3) (*n* = 205)	2 (2–3) (*n* = 209)	3 (2–3) (*n* = 147)	0.223
Number of blastocysts in IVF cycle (*n* = 928)	4 (3–6) (*n* = 284)	4 (3–6) (*n* = 418)	4 (3–6) (*n* = 226)	0.495
Endometrial thickness on trigger day (mm)	11.2 (9.7–12.7)	11.1 (9.5–13.0)	11.2 (9.5–13.1)	0.755
Stage of embryos in frozen transfer				0.010*
Cleavage stage embryos	205 (41.9)	209 (33.3)	147 (39.4)	
Blastocysts	284 (58.1)	418 (66.7)	226 (60.6)	
Blastulation rate (only for women undergoing extended culture)	50.0% (33.3%–66.7%) (*n* = 284)	48.5% (33.3%–63.6%) (*n* = 418)	50.0% (33.3%–66.7%) (*n* = 226)	0.252
Number of embryos transferred				0.601
1	203 (99.0)	207 (99.0)	144 (98.0)	
2	2 (1.0)	2 (1.0)	3 (2.0)	
Number of blastocysts transferred				–
1	284 (100.0)	418 (100.0)	226 (100.0)	
Endometrial preparation				0.044*
Natural cycle	310 (63.4)	353 (56.3)	233 (62.5)	
Hormonal replacement cycle	116 (23.7)	189 (30.1)	103 (27.6)	
Letrozole	63 (12.9)	85 (13.6)	36 (9.7)	
Gonadotrophin stimulation	0 (0.0)	0 (0.0)	1 (0.1)	

Data shown represent the median (25th–75th percentile) for continuous variables and number (%) for categorical variables

^*^Statistically significant

^#^Kruskal–Wallis test for continuous variables

## Discussion

Our study showed that serum vitamin D level was not associated with the live birth rate in women undergoing frozen embryo transfer. There were no statistically significant differences in the pregnancy rates, ongoing pregnancy rates, and miscarriage rates between vitamin D deficient and non-deficient groups. However, more women in the vitamin D non-deficient group had blastocyst transfer than cleavage stage embryo transfer compared to the vitamin D deficient group.

We have previously published a retrospective study at our unit which found that the cumulative live birth rate of the first IVF cycle, including the fresh and all frozen embryo transfers from one stimulated IVF cycle, in the vitamin D deficient group was significantly lower compared to the non-deficient group [15]. In that study, women with vitamin D deficiency had a higher body mass index and had less oocytes retrieved and normally fertilized oocytes despite requiring a higher dosage of gonadotrophin for ovarian stimulation compared to the vitamin D non-deficient group, although the magnitude of absolute difference was small. The usual practice in our unit is for extended culture to blastocyst if there are more than 3 cleavage-stage embryos. In our current study on frozen embryo transfer, the dosage of gonadotrophins stimulation, number of oocytes retrieved and number of normally fertilized oocytes in the index IVF cycle were similar in the vitamin D deficient and non-deficient groups. Taking the two studies together, one hypothesis is that vitamin D may act on enhancing oocyte or embryo quality by improving blastocyst formation rather than on endometrial receptivity in affecting pregnancy outcome. However, there were no differences in the blastulation rate between the vitamin D deficient and non-deficient groups when only women with extended culture to blastocysts were considered. We did not find statistically significant differences in endometrial thickness between the vitamin D deficient and non-deficient groups.

Our findings are in contrast to one study of 368 infertile women undergoing single blastocyst transfer which showed that vitamin D deficiency compromised pregnancy rates by 40%, but similar to 2 recent studies on the association of vitamin D and frozen embryo transfer which did not find serum vitamin D status to be related to pregnancy outcomes [21, 22]. In the study by Franasiak et al., only women with euploid blastocyst transfers were included to control for embryonic factor, although both fresh or frozen embryo transfers were included and the results may not be applicable to women who did not undergo extended embryo culture or preimplantation genetic testing for euploid blastocyst transfer [21]. A prospective observational cohort study by van de Vijver et al. included 280 women who had 1–2 blastocyst(s) transferred [22]. In these studies, the rationale of including only blastocyst transfers or even euploid blastocysts was to minimize the biases related to oocyte and embryo quality and actually evaluate whether vitamin D deficiency independently compromises the chances of pregnancy in those reaching the blastocyst stage. However, this would imply the inclusion of a selected ‘better prognosis’ group. We included a larger sample size of women from two tertiary reproductive medicine centres undergoing frozen embryo transfer. Our study population was more heterogenous and included all women who had frozen embryo transfer during the study period, resulting in a mix of women undergoing cleavage stage embryos and blastocyst transfer, but may be more generalizable to the general infertile population. Most (99.5%) of the women in our study had single cleavage-stage embryo or blastocyst transfer. We included the first frozen embryo transfer in our centre as it is generally the practice that better-quality embryos are transferred first. However, we may not be able to entirely exclude all women who may have had embryo transfers or previous IVF cycles prior to the study period.

Existing studies on vitamin D and frozen embryo transfer involved mostly Caucasian population and serum vitamin D was assessed using immunoassay [20–22]. A retrospective study by Rudick et al. suggested that the relationship between vitamin D status and pregnancy rates differed by race, with vitamin D deficiency leading to lower pregnancy rates in Caucasian women, but the opposite in Asians [26]. They cautioned that results from their study need to be validated by future cohort studies, given that only 49 out of 188 women were of Asian origin. In another study by the same group on recipients of donor oocytes, which found a lower pregnancy rate in recipients of egg donation who were vitamin D deficient compared to the vitamin D replete group, Asians were the only racial group for which there was no evidence of a beneficial effect of vitamin D albeit the small numbers [16]. On the contrary, the majority of our patients were Chinese, which may be an important contribution to the controversy about the association of vitamin D and FET outcomes with regard to ethnicity. However, similarly, we were not able to assess for the effect of ethnicity owing to small number of non-Chinese population. Further research is needed to elucidate racial differences in the effect of vitamin D on reproduction. In our study, serum 25(OH)D was assessed by mass spectrometry in an accredited laboratory, which is the gold standard.

In our study on frozen embryo transfer, we still found a high prevalence of vitamin D deficiency (32.8%) and insufficiency (42.1%). We adopted the most widely used classification by The Endocrine Society of serum vitamin D replete status as > 75 nmol/l (> 30 ng/ml), deficiency as < 50 nmol/l (< 20 ng/ml) and insufficiency as that in between these levels [25]. For the purpose of the main analysis, we have grouped the vitamin D insufficient and replete status together as ‘non-deficient’ state. Existing studies and meta-analysis vary in the classification of the intermediate group of ‘insufficient’ vitamin D status, some grouping it together with the replete group as in our study, while others may group it together with the deficient group [27]. When analysed as three separate groups, we still did not find statistically significant differences in the live birth rates, pregnancy rates, ongoing pregnancy rates and miscarriage rates among the three groups. There is still no consensus on the appropriate cut-off level to define vitamin D deficiency for non-skeletal effects or whether vitamin D supplementation is beneficial in these conditions [28, 29]. Vitamin D levels exist as a continuum and women with vitamin D level at very similar levels close to the cut-off may have been classified into different categories based on very small differences. There is evidence that even higher thresholds of vitamin D beyond 75 nmol/L are beneficial for reproduction in women with polycystic ovary syndrome undergoing ovulation induction [30]. Our analysis is limited by the small number of women with serum vitamin D beyond these levels.

The main limitation of our study is its retrospective and cross-sectional nature. Livebirth was our primary outcome. Indeed, there was a long way to go from frozen embryo transfer until live birth and women may be on various supplements during the course of pregnancy, which we have not documented. We also looked at the ongoing pregnancy rate and early miscarriage rate of our cohort and there were no statistically significant differences in these parameters between the vitamin D deficient and non-deficient groups. A systematic review and meta-analysis have shown an increased risk of miscarriage in naturally conceived women who were vitamin D deficient compared with those who were vitamin D replete [31]. On the contrary, vitamin D did not seem to be associated with miscarriage in several meta-analyses that included women undergoing IVF [10, 12, 27]. A recently published Mendelian randomization study also showed no evidence of a causal relationship between miscarriage and serum 25(OH)D level [32].

Concerns have been raised regarding the potential degradation of serum 25(OH)D with storage which may potentially affect interpretation in retrospective studies [33, 34]. Our prospective observational study involving 55 reproductive-aged women undergoing assisted reproductive treatment where we assessed the change in serum 25(OH)D with cryostorage showed that the median percentage decrease in serum 25(OH)D was only 3.4% (statistically not significant) when the serum samples were stored at − 20 °C for 7 months and analysed with mass spectrometry (unpublished). Nevertheless, being a retrospective study based on a convenience sample size, our study may be underpowered to assess the association of vitamin D on pregnancy rate if the effect of vitamin D was too small to be shown in this study. There was a 5% difference in ongoing pregnancy rate between the vitamin D deficient and non-deficient groups although not statistically significant. Our retrospective power analysis suggested that our current sample size would only give a power of 45% to detect a difference of 5%. Although it is arguable whether a difference of 5% is clinically meaningful, Vitamin D is a safe and cheap intervention and vitamin D deficiency can be easily corrected by dietary supplements. This small improvement in pregnancy rate may potentially be cost-effective and relevant to infertile women undergoing in IVF taken into consideration the safety of intervention. Future adequately powered interventional studies on the effect of vitamin D on the live birth rate in infertile women undergoing frozen embryo transfer are needed to answer the question on whether vitamin D supplement should be provided.

## Conclusion

Serum vitamin D level was not associated with the live birth rate in women undergoing frozen embryo transfer.

## Data Availability

The datasets generated during and/or analysed during the current study are available from the corresponding author on reasonable request. Data will be made available to the editors of the journal for review or query upon request.

## References

[1] van Schoor N, Lips P. Global overview of vitamin D status. Endocrinol Metab Clin North Am. 2017;46(4):845–70. 10.1016/j.ecl.2017.07.002. 29080639 10.1016/j.ecl.2017.07.002

[2] Holick MF. The vitamin D deficiency pandemic: approaches for diagnosis, treatment and prevention. Rev Endocr Metab Disord. 2017;18(2):153–65. 10.1007/s11154-017-9424-1. 28516265 10.1007/s11154-017-9424-1

[3] Lerchbaum E, Obermayer-Pietsch B. Vitamin D and fertility: a systematic review. Eur J Endocrinol. 2012;166(5):765–78. 10.1530/EJE-11-0984. 22275473 10.1530/EJE-11-0984

[4] Irani M, Merhi Z. Role of vitamin D in ovarian physiology and its implication in reproduction: a systematic review. Fertil Steril 2014;102(2):460–8 e3. 10.1016/j.fertnstert.2014.04.046 10.1016/j.fertnstert.2014.04.046 24933120

[5] Ashour H, Gamal SM, Sadek NB, Rashed LA, Hussein RE, Kamar SS, et al. Vitamin D supplementation improves uterine receptivity in a rat model of vitamin D deficiency: a possible role of HOXA-10/FKBP52 axis. Front Physiol. 2021;12:744548. 10.3389/fphys.2021.744548. 34899377 10.3389/fphys.2021.744548 PMC8655728

[6] Guo J, Liu S, Wang P, Ren H, Li Y. Characterization of VDR and CYP27B1 expression in the endometrium during the menstrual cycle before embryo transfer: implications for endometrial receptivity. Reprod Biol Endocrinol. 2020;18(1):24. 10.1186/s12958-020-00579-y. 32183826 10.1186/s12958-020-00579-y PMC7079352

[7] Zehnder D, Evans KN, Kilby MD, Bulmer JN, Innes BA, Stewart PM, et al. The ontogeny of 25-hydroxyvitamin D(3) 1alpha-hydroxylase expression in human placenta and decidua. Am J Pathol. 2002;161(1):105–14. 10.1016/s0002-9440(10)64162-4. 12107095 10.1016/s0002-9440(10)64162-4 PMC1850695

[8] Chan SY, Susarla R, Canovas D, Vasilopoulou E, Ohizua O, McCabe CJ, et al. Vitamin D promotes human extravillous trophoblast invasion in vitro. Placenta. 2015;36(4):403–9. 10.1016/j.placenta.2014.12.021. 25596923 10.1016/j.placenta.2014.12.021

[9] Zhao J, Huang X, Xu B, Yan Y, Zhang Q, Li Y. Whether vitamin D was associated with clinical outcome after IVF/ICSI: a systematic review and meta-analysis. Reproduct Biol Endocrinol: RB&E. 2018;16(1):13. 10.1186/s12958-018-0324-3. 10.1186/s12958-018-0324-3 PMC5807754 29426322

[10] Chu J, Gallos I, Tobias A, Tan B, Eapen A, Coomarasamy A. Vitamin D and assisted reproductive treatment outcome: a systematic review and meta-analysis. Hum Reprod. 2018;33(1):65–80. 10.1093/humrep/dex326. 29149263 10.1093/humrep/dex326

[11] Iliuta F, Pijoan JI, Lainz L, Exposito A, Matorras R. Women's vitamin D levels and IVF results: a systematic review of the literature and meta-analysis, considering three categories of vitamin status (replete, insufficient and deficient). Hum Fertil (Camb) 2020:1–19. 10.1080/14647273.2020.1807618 10.1080/14647273.2020.1807618 32791871

[12] Cozzolino M, Busnelli A, Pellegrini L, Riviello E, Vitagliano A. How vitamin D level influences in vitro fertilization outcomes: results of a systematic review and meta-analysis. Fertil Steril. 2020;114(5):1014–25. 10.1016/j.fertnstert.2020.05.040. 33012554 10.1016/j.fertnstert.2020.05.040

[13] Tsuprykov O, Chen X, Hocher CF, Skoblo R, Lianghong Y, Hocher B. Why should we measure free 25(OH) vitamin D? J Steroid Biochem Mol Biol. 2018;180:87–104. 10.1016/j.jsbmb.2017.11.014. 29217467 10.1016/j.jsbmb.2017.11.014

[14] Mizrachi Y, Horowitz E, Farhi J, Raziel A, Weissman A. Ovarian stimulation for freeze-all IVF cycles: a systematic review. Hum Reprod Update. 2020;26(1):118–35. 10.1093/humupd/dmz037. 31867625 10.1093/humupd/dmz037

[15] Ko JKY, Shi J, Li RHW, Yeung WSB, Ng EHY. 100 years of vitamin D: Effect of serum vitamin D level before ovarian stimulation on the cumulative live birth rate of women undergoing in vitro fertilization: a retrospective analysis. Endocr Connect. 2022;11(2). 10.1530/EC-21-0444 10.1530/EC-21-0444 PMC8859949 35029541

[16] Rudick BJ, Ingles SA, Chung K, Stanczyk FZ, Paulson RJ, Bendikson KA. Influence of vitamin D levels on in vitro fertilization outcomes in donor-recipient cycles. Fertil Steril. 2014;101(2):447–52. 10.1016/j.fertnstert.2013.10.008. 24210230 10.1016/j.fertnstert.2013.10.008

[17] Fabris AM, Cruz M, Iglesias C, Pacheco A, Patel A, Patel J, et al. Impact of vitamin D levels on ovarian reserve and ovarian response to ovarian stimulation in oocyte donors. Reprod Biomed Online. 2017;35(2):139–44. 10.1016/j.rbmo.2017.05.009. 28625761 10.1016/j.rbmo.2017.05.009

[18] Fabris A, Pacheco A, Cruz M, Puente JM, Fatemi H, Garcia-Velasco JA. Impact of circulating levels of total and bioavailable serum vitamin D on pregnancy rate in egg donation recipients. Fertil Steril. 2014;102(6):1608–12. 10.1016/j.fertnstert.2014.08.030. 25256926 10.1016/j.fertnstert.2014.08.030

[19] Banker M, Sorathiya D, Shah S. Vitamin D deficiency does not influence reproductive outcomes of IVF-ICSI: a study of oocyte donors and recipients. J Hum Reprod Sci. 2017;10(2):79–85. 10.4103/jhrs.JHRS_117_16. 28904494 10.4103/jhrs.JHRS_117_16 PMC5586094

[20] Polyzos NP, Anckaert E, Guzman L, Schiettecatte J, Van Landuyt L, Camus M, et al. Vitamin D deficiency and pregnancy rates in women undergoing single embryo, blastocyst stage, transfer (SET) for IVF/ICSI. Hum Reprod. 2014;29(9):2032–40. 10.1093/humrep/deu156. 24951484 10.1093/humrep/deu156

[21] Franasiak JM, Molinaro TA, Dubell EK, Scott KL, Ruiz AR, Forman EJ, et al. Vitamin D levels do not affect IVF outcomes following the transfer of euploid blastocysts. Am J Obstet Gynecol 2015;212(3):315 e1–6. 10.1016/j.ajog.2014.09.029 10.1016/j.ajog.2014.09.029 25265402

[22] van de Vijver A, Drakopoulos P, Van Landuyt L, Vaiarelli A, Blockeel C, Santos-Ribeiro S, et al. Vitamin D deficiency and pregnancy rates following frozen-thawed embryo transfer: a prospective cohort study. Hum Reprod. 2016;31(8):1749–54. 10.1093/humrep/dew107. 27170434 10.1093/humrep/dew107

[23] Yung SSF, Lai SF, Lam MT, Lui EMW, Ko JKY, Li HWR, et al. Hyaluronic acid-enriched transfer medium for frozen embryo transfer: a randomized, double-blind, controlled trial. Fertil Steril. 2021;116(4):1001–9. 10.1016/j.fertnstert.2021.02.015. 33845988 10.1016/j.fertnstert.2021.02.015

[24] Chen ZQ, Ai A, Zhang Y, Li H, Wang JY, Wang L, et al. A randomized controlled trial to compare the live birth rate of the first frozen embryo transfer following the progestin-primed ovarian stimulation protocol vs. the antagonist protocol in women with an anticipated high ovarian response. Fertil Steril. 2024. 10.1016/j.fertnstert.2024.01.027 10.1016/j.fertnstert.2024.01.027 38272383

[25] Holick MF, Binkley NC, Bischoff-Ferrari HA, Gordon CM, Hanley DA, Heaney RP, et al. Evaluation, treatment, and prevention of vitamin D deficiency: an endocrine society clinical practice guideline. J Clin Endocrinol Metab. 2011;96(7):1911–30. 10.1210/jc.2011-0385. 21646368 10.1210/jc.2011-0385

[26] Rudick B, Ingles S, Chung K, Stanczyk F, Paulson R, Bendikson K. Characterizing the influence of vitamin D levels on IVF outcomes. Hum Reprod. 2012;27(11):3321–7. 10.1093/humrep/des280. 22914766 10.1093/humrep/des280

[27] Iliuta F, Pijoan JI, Lainz L, Exposito A, Matorras R. Women’s vitamin D levels and IVF results: a systematic review of the literature and meta-analysis, considering three categories of vitamin status (replete, insufficient and deficient). Hum Fertil (Camb). 2022;25(2):228–46. 10.1080/14647273.2020.1807618. 32791871 10.1080/14647273.2020.1807618

[28] Autier P, Mullie P, Macacu A, Dragomir M, Boniol M, Coppens K, et al. Effect of vitamin D supplementation on non-skeletal disorders: a systematic review of meta-analyses and randomised trials. Lancet Diabetes Endocrinol. 2017;5(12):986–1004. 10.1016/S2213-8587(17)30357-1. 29102433 10.1016/S2213-8587(17)30357-1

[29] Giustina A, Adler RA, Binkley N, Bollerslev J, Bouillon R, Dawson-Hughes B, et al. Consensus statement from 2(nd) International Conference on Controversies in Vitamin D. Rev Endocr Metab Disord 2020;21(1):89–116. 10.1007/s11154-019-09532-w 10.1007/s11154-019-09532-w PMC7113202 32180081

[30] Pal L, Zhang H, Williams J, Santoro NF, Diamond MP, Schlaff WD, et al. Vitamin D status relates to reproductive outcome in women with polycystic ovary syndrome: secondary analysis of a multicenter randomized controlled trial. J Clin Endocrinol Metab. 2016;101(8):3027–35. 10.1210/jc.2015-4352. 27186859 10.1210/jc.2015-4352 PMC4971341

[31] Tamblyn JA, Pilarski NSP, Markland AD, Marson EJ, Devall A, Hewison M, et al. Vitamin D and miscarriage: a systematic review and meta-analysis. Fertil Steril. 2022;118(1):111–22. 10.1016/j.fertnstert.2022.04.017. 35637024 10.1016/j.fertnstert.2022.04.017

[32] Zhang F, Huang J, Zhang G, Dai M, Yin T, Huang C, et al. No evidence of a causal relationship between miscarriage and 25-hydroxyvitamin D: a Mendelian randomization study. Hum Reprod Open. 2024;2024(2):hoae011. 10.1093/hropen/hoae011 10.1093/hropen/hoae011 PMC10918637 38456064

[33] Klimczak AM, Franasiak JM. Vitamin D in human reproduction: some answers and many more questions. Fertil Steril. 2021;115(3):590–1. 10.1016/j.fertnstert.2020.12.027. 33516577 10.1016/j.fertnstert.2020.12.027

[34] Lara-Molina E, Franasiak J, Devesa-Peiro A, Lopez-Nogueroles M, Florensa M, Martin M, et al. The degradation of vitamin D across time: an issue leading to unreliable results in reproductive research. Fertil Steril. 2019;112(suppl):E339–40.

